# Survival, Dependency, and Health-Related Quality of Life in Patients With Ruptured Intracranial Aneurysm: 10-Year Follow-up of the United Kingdom Cohort of the International Subarachnoid Aneurysm Trial

**DOI:** 10.1093/neuros/nyaa454

**Published:** 2020-10-19

**Authors:** Xinyang Hua, Alastair Gray, Jane Wolstenholme, Philip Clarke, Andrew J Molyneux, Richard S C Kerr, Alison Clarke, Mary Sneade, Oliver Rivero-Arias

**Affiliations:** National Perinatal Epidemiology Unit, Nuffield Department of Population Health, University of Oxford, Oxford, United Kingdom; Health Economics Research Centre, Nuffield Department of Population Health, University of Oxford, Oxford, United Kingdom; Health Economics Research Centre, Nuffield Department of Population Health, University of Oxford, Oxford, United Kingdom; Health Economics Research Centre, Nuffield Department of Population Health, University of Oxford, Oxford, United Kingdom; Nuffield Department of Surgical Sciences, University of Oxford, Oxford, United Kingdom; Department of Neurosurgery, John Radcliffe Hospital, Oxford, United Kingdom; Nuffield Department of Surgical Sciences, University of Oxford, Oxford, United Kingdom; Nuffield Department of Surgical Sciences, University of Oxford, Oxford, United Kingdom; National Perinatal Epidemiology Unit, Nuffield Department of Population Health, University of Oxford, Oxford, United Kingdom

**Keywords:** Endovascular coiling, Quality of life, Randomized controlled trail, Subarachnoid hemorrhage

## Abstract

**BACKGROUND:**

Previous analyses of the International Subarachnoid Aneurysm Trial (ISAT) cohort have reported on clinical outcomes after treatment of a ruptured intracranial aneurysm with either neurosurgical clipping or endovascular coiling.

**OBJECTIVE:**

To evaluate the long-term quality-adjusted life years (QALYs) gained of endovascular coiling compare to neurosurgical clipping in the UK cohort of ISAT.

**METHODS:**

Between September 12, 1994 and May 1, 2002, patients with ruptured intracranial aneurysms who were assumed treatment equipoise were randomly allocated to either neurosurgical clipping or endovascular coiling. We followed-up 1644 patients in 22 UK neurosurgical centers for a minimum of 10 yr. Health-related quality of life (HRQoL) was collected through yearly questionnaires, measured by utilities calculated from the EQ-5D-3L. We compared HRQoL between the 2 treatment groups over a period of 10 yr. In all, 1-yr, 5-yr, and 10-yr QALYs were estimated by combining utility and survival information.

**RESULTS:**

Higher average utility values were found in the endovascular group throughout the follow-up period, with mean differences between groups statistically significant in most years. The 10-yr QALYs were estimated to be 6.68 (95% CI: 6.45-6.90) in the coiling group and 6.32 (95% CI: 6.10-6.55) in the clipping group, respectively, a significant mean difference of 0.36 (95% CI: 0.04-0.66). A third of this mean QALYs gain was estimated to derive solely from HRQoL differences.

**CONCLUSION:**

HRQoL after treatment of a ruptured intracranial aneurysm was better after endovascular coiling compared to neurosurgical clipping, which contributed significantly to the QALYs gained over a 10-yr period.

## Graphical Abstract

**Graphical Abstract:**
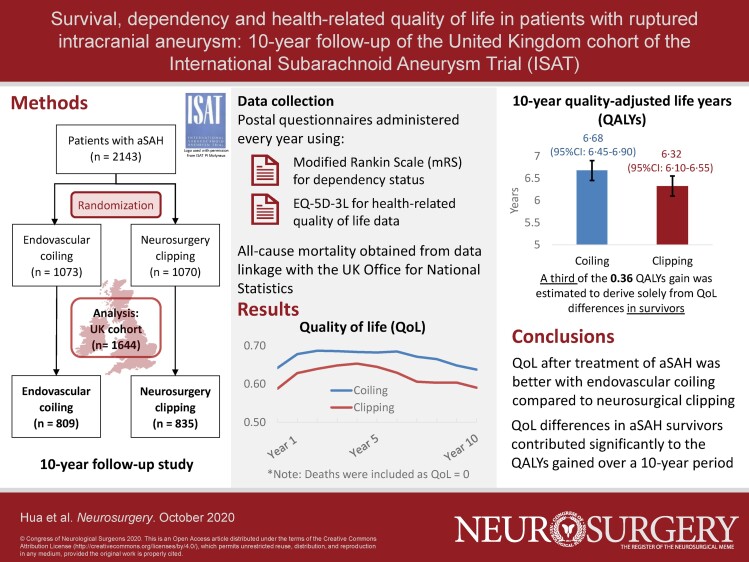

**ABBREVIATIONS**
CI: confidence interval
CONSORT: Consolidated Standards of Reporting Trials
HRQoL: health-related quality of life
ISAT: International Subarachnoid Aneurysm Trial
mRS: modified Rankin Scale
OR: odds ratio
QALYs: quality-adjusted life years
SAH: subarachnoid hemorrhage
SD: standard deviation

The chance of a patient surviving an aneurysmal subarachnoid hemorrhage (SAH) was estimated to have increased largely in the past few decades.^1-5^ A large proportion of the survivors from a SAH would suffer a functional and cognitive impairment and difficulty on activities of daily living (eg, feeding, dressing, and bathing), which affects patient's health-related quality of life (HRQoL).^6,7^

Endovascular coiling is now recommended as a more preferred treatment than neurosurgical clipping in aneurysmal SAH patients who are considered equally suitable for either therapeutic option,^8^ mainly based on findings from the International Subarachnoid Aneurysm Trial (ISAT), which suggested that coiling resulted in significantly fewer dead and dependent patients.^9-12^ However, besides the effects on clinical outcomes, there is currently no robust evidence on the impact of coiling and clipping on long-term HRQoL. Previous studies on this topic were mostly conducted in nonrandom fashion with a small sample size, which leads to inconsistent results.^13-15^

In this study, we reported the HRQoL outcomes from the UK cohort of ISAT and evaluated trends in HRQoL over 10 yr in the endovascular coiling group and the neurosurgical clipping group. Dependency was also revisited in each year using the full ordinal range of the modified Rankin Scale (mRS) instead of a dichotomous variable as presented in earlier work.^11^ We estimated and compared the overall effectiveness of the treatments by calculating life years and quality-adjusted life years (QALYs) gained over a 10-yr period.

## METHODS

### Patients and Procedures

The aim of ISAT was to compare the safety and efficacy of endovascular treatment vs conventional neurosurgical treatment of ruptured intracranial aneurysms in patients who were suitable for either treatment. Between September 12, 1994 and May 1, 2002, 2143 patients with ruptured intracranial aneurysms with small anterior circulation aneurysms in good grade (90% grade 1 and 2), mostly from the UK, the rest of Europe, North America, and Australia, were enrolled into ISAT and randomly allocated to either neurosurgical clipping (n = 1070) or endovascular coiling (n = 1073). The detailed methods of the trial and patient inclusion and exclusion criteria have been described in the trial protocol.^9^ This study was based on the UK cohort of ISAT (n = 1644), which was enrolled from 22 participating neurosurgical centers that provided both neurosurgical and endovascular treatment for acute SAH from ruptured aneurysms.

Between the date of enrolment and May 2012, 2 mo after the intervention and annually, all surviving ISAT patients in the UK were sent a postal questionnaire that assessed their dependency status and HRQoL. If the patient was unable to complete the questionnaire, we allowed proxy responses from carers, as this has previously been shown to be valid in this context.^16^ All UK patients were flagged with the Office for National Statistics. The Oxford Neurovascular and Neuroradiology Research Unit automatically received notification of the death of patients registered in the study.

### Outcomes

We assessed rates of death, dependence, and HRQoL after neurosurgical clipping or endovascular coiling over a period of 10 yr. Dependency status was measured by the patient-reported mRS questionnaire,^17^ and HRQoL was measured by the 3-level version of EQ-5D (EQ-5D-3L) questionnaire developed by the EuroQol Group^18^ (**Text, Supplemental Digital Content 1**).

### Statistical Analysis

We presented the distribution of the mRS and the distribution of each EQ-5D-3L domain, as well as the mean utility values generated from the EQ-5D-3L, for the endovascular coiling and neurosurgery clipping group at 2 mo and annually up to 10 yr. Chi-square test was used to examine differences in the distribution of responses to the mRS and of each EQ-5D-3L domain between the 2 groups. Parametric Student t-test was used to examine differences in EQ-5D-3L utility scores between groups. Dead cases were identified in this descriptive analysis as “level 6” for the mRS and “0” for utility score. Adjustments to missing values mRS and EQ-5D-3L responses were made (**Text, Supplemental Digital Content 2**).

We also compared the mRS and the EQ-5D-3L utility scores between the 2 groups only using SAH survivors (excluding deaths) to capture potential treatment effect on dependency and quality of life (HRQoL) of endovascular coiling over neurosurgery clipping beyond survival.

QALYs were estimated as product of the mean utility score for SAH survivors at each follow-up points (2-mo and annually after) and the corresponding mean life years at that period from a multivariate Cox model fitted for all-cause mortality (**Text, Supplemental Digital Content 3**). Detailed step-by-step methods to estimate life years and QALYs are presented in **Text, Supplemental Digital Content 4**. Mean 1-yr, 5-yr, and 10-yr life years and QALYs were estimated for the whole cohort and for the endovascular coiling group and neurosurgery clipping group separately, reported alongside differences between the 2 groups with 95% CIs. The 10-year mean difference in QALYs was decomposed into a contribution from survival and HRQoL (**Text, Supplemental Digital Content 4**).

A subgroup analysis was conducted to examine the difference in life years and QALYs between the 2 treatment groups among patients with different baseline characteristics (sex, World Federation of Neurological Surgeons grade, maximum target aneurysm lumen size, and number of aneurysms detected).

The last available value carried forward method was applied in the main ISAT paper to fill in missing mRS scores and was shown to produce similar results compared with using complete cases.^11^ We used complete cases in the main analysis and conducted a scenario analysis by applying the same method for missing utility values to calculate QALYs.

### Standard Protocol Approvals, Registrations, and Patient Consents

All UK centers obtained local ethical committee approval for the study before enrolling patients. Appropriate consent of the patient or assent of relatives was obtained for all study participants. ISAT and the publication of its results, the study has adhered to the strict standards and procedures for the conduct, design, and reporting of clinical trials, as laid out in the Consolidated Standards of Reporting Trials (CONSORT) guidelines and its Extension for Patient-Reported Outcomes (see **Figure, Supplemental Digital Content 5** for the CONSORT flow chart). ISAT is registered, number ISRCTN49866681.

## RESULTS

Baseline characteristics of this UK cohort from ISAT are reported in Table 1. The 1644 patients were followed-up with a median of 12.8 yr (19 215.8 patient-years in total). At year 10, 1331 of the 1644 patients had survived, among which 1023 (77%) and 980 (74%) patients had a complete report on the mRS and the EQ-5D-3L, respectively. Patients with missing data in years 5 and 10 were significantly younger in both groups compared with patients with a complete report (**Tables, Supplemental Digital Contents 6-9**). We also observed marginally more missing data in the neurosurgical group compared to endovascular coiling at 2-mo and 5- and 10-yr follow-up (**Figure, Supplemental Digital Content 5**), but the baseline characteristics of missing data patients between both groups were similar.

**TABLE 1.** Baseline Characteristics for UK Patients (n = 1644)

	Endovascular coiling (n = 809)	Neurosurgery clipping (n = 835)
Age (years)*	51 (44-60, 18-87)	52 (44-60, 20-84)
Sex		
Female	517 (64%)	530 (64%)
Male	292 (36%)	305 (37%)
WFNS grade		
1	546 (67%)	546 (65%)
2	185 (23%)	212 (25%)
3	50 (6%)	55 (7%)
4	18 (2%)	16 (2%)
5	5 (1%)	0 (0%)
6 (not assessable)	5 (1%)	6 (1%)
Maximum target aneurysm lumen size (mm)		
≤5	431 (53%)	451 (54%)
6-10	322 (40%)	328 (39%)
≥11	56 (7%)	56 (7%)
Number of aneurysms detected		
1	615 (76%)	650 (77%)
2	145 (18%)	139 (17%)
3	32 (4%)	32 (4%)
≥4	17 (2%)	14 (2%)
Time between SAH and randomization (days)*	2 (1-5, 0-26)	3 (1-6, 0-28)

WFNS = World Federation of Neurological Surgeons.

*Median (lower quartile - upper quartile, range).

We found statistically significant differences (*P*-value < .05) on the distribution of the mRS between the 2 treatment groups in all the follow-up time points from 2 mo to 10 yr after the intervention, with a consistent higher proportion reporting “no symptoms” (mRS level 0) and a lower proportion of death (mRS level 6) in the endovascular group (Table 2). The proportion reporting “minor symptoms” (mRS level 1) was also found higher in the endovascular group in almost all follow-up years (except for year 5, in which the proportions were similar) (Table 2). Among SAH survivors, the difference in the distribution of the mRS between the 2 treatment groups was also found to be statistically significant in most follow-up years including year 10 (**Table, Supplemental Digital Content 10**), indicating a better independence status in the endovascular group.

**TABLE 2.** Ten-Year Follow-up of Survival and mRS, by Treatment Groups

	Endovascular coiling (n = 809)			Neurosurgery clipping (n = 835)				
	mRS	No.	Percentage*	mRS	No.	Percentage*	Difference in percentage	Chi-square for mRS (*P* value)^†^
2 mo	0	141	17.5%	0	103	12.4%	5.1%	34.47
	1	243	30.1%	1	213	25.6%	4.5%	(<.001)
	2	218	27.0%	2	199	24.0%	3.1%	
	3	94	11.6%	3	163	19.6%	−8.0%	
	4	22	2.7%	4	36	4.3%	−1.6%	
	5	42	5.2%	5	50	6.0%	−0.8%	
	6 (Dead)	47	5.8%	6 (Dead)	67	8.0%	−2.2%	
Year 1	0	188	23.3%	0	144	17.4%	5.9%	19.01
	1	231	28.6%	1	228	27.5%	1.1%	(.004)
	2	193	23.9%	2	189	22.8%	1.1%	
	3	98	12.1%	3	120	14.5%	−2.3%	
	4	25	3.1%	4	37	4.5%	−1.4%	
	5	16	2.0%	5	29	3.5%	−1.5%	
	6 (Dead)	56	6.9%	6 (Dead)	83	9.9%	−3.0%	
Year 2	0	228	28.8%	0	193	24.4%	4.4%	16.89
	1	214	27.1%	1	191	24.1%	2.9%	(.010)
	2	168	21.2%	2	165	20.9%	0.4%	
	3	76	9.6%	3	110	13.9%	−4.3%	
	4	28	3.5%	4	29	3.7%	−0.1%	
	5	20	2.5%	5	17	2.1%	0.4%	
	6 (Dead)	58	7.2%	6 (Dead)	91	10.9%	−3.7%	
Year 3	0	231	30.0%	0	183	23.7%	6.3%	21.069
	1	208	27.0%	1	191	24.8%	2.2%	(.002)
	2	149	19.4%	2	168	21.8%	−2.4%	
	3	78	10.1%	3	100	13.0%	−2.8%	
	4	26	3.4%	4	17	2.2%	1.2%	
	5	17	2.2%	5	14	1.8%	0.4%	
	6 (Dead)	64	7.9%	6 (Dead)	106	12.7%	−4.8%	
Year 4	0	228	30.1%	0	191	25.4%	4.7%	13.08
	1	196	25.9%	1	175	23.3%	2.6%	(.042)
	2	141	18.6%	2	156	20.7%	−2.1%	
	3	85	11.2%	3	95	12.6%	−1.4%	
	4	23	3.0%	4	19	2.5%	0.5%	
	5	17	2.2%	5	16	2.1%	0.1%	
	6 (Dead)	71	8.8%	6 (Dead)	111	13.3%	−4.5%	
Year 5	0	245	33.1%	0	181	24.4%	8.7%	21.43
	1	182	24.6%	1	188	25.3%	−0.8%	(.002)
	2	122	16.5%	2	151	20.3%	−3.9%	
	3	80	10.8%	3	87	11.7%	−0.9%	
	4	24	3.2%	4	20	2.7%	0.5%	
	5	18	2.4%	5	12	1.6%	0.8%	
	6 (Dead)	76	9.4%	6 (Dead)	116	13.9%	−4.5%	
Year 6	0	232	32.8%	0	175	24.6%	8.2%	23.96
	1	180	25.5%	1	155	21.8%	3.6%	(.001)
	2	118	16.7%	2	160	22.5%	−5.8%	
	3	64	9.1%	3	81	11.4%	−2.3%	
	4	19	2.7%	4	20	2.8%	−0.1%	
	5	17	2.4%	5	13	1.8%	0.6%	
	6 (Dead)	88	10.9%	6 (Dead)	125	15.0%	−4.1%	
Year 7	0	240	34.3%	0	184	26.3%	8.0%	22.45
	1	168	24.0%	1	149	21.3%	2.7%	(.001)
	2	106	15.1%	2	132	18.9%	−3.7%	
	3	62	8.9%	3	79	11.3%	−2.4%	
	4	17	2.4%	4	24	3.4%	−1.0%	
	5	18	2.6%	5	10	1.4%	1.1%	
	6 (Dead)	103	12.7%	6 (Dead)	145	17.4%	−4.6%	
Year 8	0	230	33.9%	0	185	26.9%	7.1%	16.95
	1	150	22.1%	1	134	19.5%	2.7%	(.009)
	2	101	14.9%	2	126	18.3%	−3.4%	
	3	73	10.8%	3	74	10.8%	0.0%	
	4	15	2.2%	4	24	3.5%	−1.3%	
	5	14	2.1%	5	14	2.0%	0.0%	
	6 (Dead)	113	14.0%	6 (Dead)	159	19.0%	−5.1%	
Year 9	0	216	32.8%	0	171	27.1%	5.7%	16.87
	1	153	23.2%	1	121	19.2%	4.1%	(.010)
	2	96	14.6%	2	116	18.4%	−3.8%	
	3	60	9.1%	3	67	10.6%	−1.5%	
	4	16	2.4%	4	22	3.5%	−1.1%	
	5	16	2.4%	5	9	1.4%	1.0%	
	6 (Dead)	125	15.5%	6 (Dead)	166	19.9%	−4.4%	
Year 10	0	227	34.7%	0	162	26.7%	8.0%	18.73
	1	137	20.9%	1	107	17.6%	3.3%	(.005)
	2	85	13.0%	2	107	17.6%	−4.6%	
	3	68	10.4%	3	75	12.3%	−2.0%	
	4	17	2.6%	4	18	3.0%	−0.4%	
	5	11	1.7%	5	9	1.5%	0.2%	
	6 (Dead)	135	16.7%	6 (Dead)	178	21.3%	−4.6%	

mRS: level 0 - no symptoms; level 1 - minor symptoms; level 2 - some restriction in lifestyle; level 3 - significant restriction in lifestyle; level 4 - partly dependent; level 5 - fully dependent; level 6 - dead.

*Percentages for mRS level 6 (dead) were calculated as number of deaths divided by the total number in the cohort; percentages for mRS level 0 to 5 were calculated by multiplying the probability of being alive and the probability of reporting on that level of mRS among patients who reported mRS.

^†^Chi-square and *P*-value were calculated based on the adjusted percentages.

A higher utility value was found in the endovascular group at all follow-up time points when deaths were included, with the difference statistically significant in most years (Table 3). For SAH survivors, the utility values were observed to be largely improved from 2 mo at year 1 (mean value of 0.73 (standard deviation [SD] 0.30) in the endovascular group vs 0.70 (SD 0.31) in the neurosurgical group, *P* = .083), and maintained a gradual increase until year 4, when it stabilized at around 0.75 to 0.76 in both groups (**Table, Supplemental Digital Content 11**). The distribution of individual EQ-5D-3L domains can be found in **Table, Supplemental Digital Content 12**. At 2 mo, patients in the endovascular group reported significantly less problems on mobility, self-care, and pain.

**TABLE 3.** Ten-Year Follow-up on HRQoL, by Treatment Groups

	Endovascular coiling (n = 809)		Neurosurgery clipping (n = 835)		T-test
	No.	EQ-5D-3L utility* Mean (SD)	No.	EQ-5D-3L utility* Mean (SD)	(*P* value)
2 mo	718	0.64 (0.34)	716	0.59 (0.36)	2.95 (.003)
Year 1	772	0.68 (0.34)	772	0.63 (0.36)	2.77 (.006)
Year 2	728	0.69 (0.35)	722	0.64 (0.36)	2.56 (.011)
Year 3	708	0.69 (0.35)	701	0.65 (0.36)	2.00 (.045)
Year 4	704	0.68 (0.36)	697	0.65 (0.37)	1.58 (.113)
Year 5	692	0.68 (0.36)	689	0.64 (0.38)	1.91 (.056)
Year 6	660	0.69 (0.37)	667	0.63 (0.38)	2.78 (.005)
Year 7	679	0.67 (0.38)	655	0.61 (0.40)	3.15 (.002)
Year 8	667	0.67 (0.38)	677	0.60 (0.40)	2.98 (.003)
Year 9	652	0.65 (0.39)	646	0.60 (0.40)	2.12 (.034)
Year 10	653	0.64 (0.39)	640	0.59 (0.41)	2.19 (.029)

*Deaths were included as utility equals to zero.

Compared with patients in the endovascular group, patients in the neurosurgery group were found to have a higher risk of all-cause mortality with a hazard ratio of 1.35 (95% CI: 1.08, 1.68) (Table 4). Patients in the endovascular coiling group were estimated to have significantly higher life years and QALYs in both the short term (1 yr) and the long term (5 and 10 yr) (Table 5). The 10-yr life years were estimated to be 8.92 (95% CI: 8.74, 9.09) in the endovascular group, compared with 8.60 (95% CI: 8.41, 8.80) in the neurosurgical group. The 10-yr QALYs were estimated to be 6.68 (95% CI: 6.45, 6.90) and 6.32 (95% CI: 6.10, 6.55) in the 2 groups, respectively, with a statistically significant difference of 0.36 (95% CI: 0.04, 0.66) yr. When using the whole cohort mean utility values instead of the treatment group specific ones to calculate the QALYs, the 10-yr difference in QALYs between the 2 groups was estimated to be 0.24 (95% CI: 0.05-0.42) yr, indicating that one-third of the 0.36 10-yr QALYs differences between the 2 groups came from the differences in HRQoL alone. In the scenario analysis in which the last value carried forward method was used to fill in the missing utility values, the 10-yr difference in QALYs between the 2 groups was 0.36 (95% CI: 0.05, 0.65), which was almost identical to the results generated by using complete cases.

**TABLE 4.** Hazard Ratios of Treatment Group and Other Patient Characteristics on All-Cause Mortality, Based on a Multivariable Cox Model*

	Hazard ratio	*P* value	95% CI
Age (year)	1.06	<.001	(1.05, 1.08)
Female	0.80	.065	(0.63, 1.01)
Neurosurgery clipping (compared to coiling)	1.35	.009	(1.08, 1.68)
WFNS grade (compared to grade 1)			
Grade 2	1.62	<.001	(1.25, 2.08)
Grade 3	1.47	.070	(0.97, 2.22)
Grades 4-6^†^	5.69	<.001	(3.74, 8.66)
Maximum target aneurysm lumen size (mm) (compared to < = 5)			
6-10	1.30	.028	(1.03, 1.65)
> = 11	1.71	.005	(1.17, 2.49)
Number of aneurysms detected (compared to 1)			
2	1.27	.099	(0.96, 1.67)
3	1.43	.171	(0.86, 2.38)
4	1.75	.101	(0.90, 3.43)

WFNS = World Federation of Neurological Surgeons.

*No interaction between the treatment and other covariates was detected; the PH assumption was examined and confirmed using Schoenfeld residuals.

^†^The hazard ratios for WFNS grade 4 to 6 were close to each other without statistically significant differences. Due to small sample size for patients with WFNS grade 4 to 6, these 3 levels were combined in the Cox model.

**TABLE 5.** Estimated 1-yr, 5-yr, and 10-yr Life Years and QALYs, by Treatment Groups

	1-yr		5-yr		10-yr	
	Mean	95% CI	Mean	95% CI	Mean	95% CI
**Life years**						
Total	0.93	(0.92, 0.94)	4.53	(4.47, 4.60)	8.76	(8.62, 8.90)
By treatment group						
Endovascular	0.94	(0.93, 0.95)	4.59	(4.52, 4.67)	8.92	(8.74, 9.09)
Neurosurgery	0.92	(0.91, 0.94)	4.47	(4.38, 4.56)	8.60	(8.41, 8.80)
Difference	0.02	(0.00, 0.03)	0.12	(0.02, 0.22)	0.32	(0.06, 0.57)
**QALYs**						
Total	0.64	(0.62, 0.65)	3.30	(3.22, 3.38)	6.50	(6.34, 6.66)
By treatment group						
Endovascular	0.66	(0.64, 0.68)	3.37	(3.27, 3.48)	6.68	(6.45, 6.90)
Neurosurgery	0.61	(0.59, 0.64)	3.23	(3.12, 3.34)	6.32	(6.10, 6.55)
Difference	0.05	(0.02, 0.08)	0.14	(0.00, 0.28)	0.36	(0.04, 0.66)

### Subgroup Analysis

Figure displays the mean differences in estimated life years and QALYs between the 2 treatment groups in different patient subgroups. The life years were estimated to be higher in the endovascular coiling group in all patient subgroups, although in a few subgroups the CI of the differences covered zero. More uncertainty was found in the QALYs differences, especially for more severe patient subgroups where the sample size was relatively small. Although not always significant, the estimated QALYs tend to be higher in the endovascular group among most patient subgroups (Figure).

**FIGURE.**
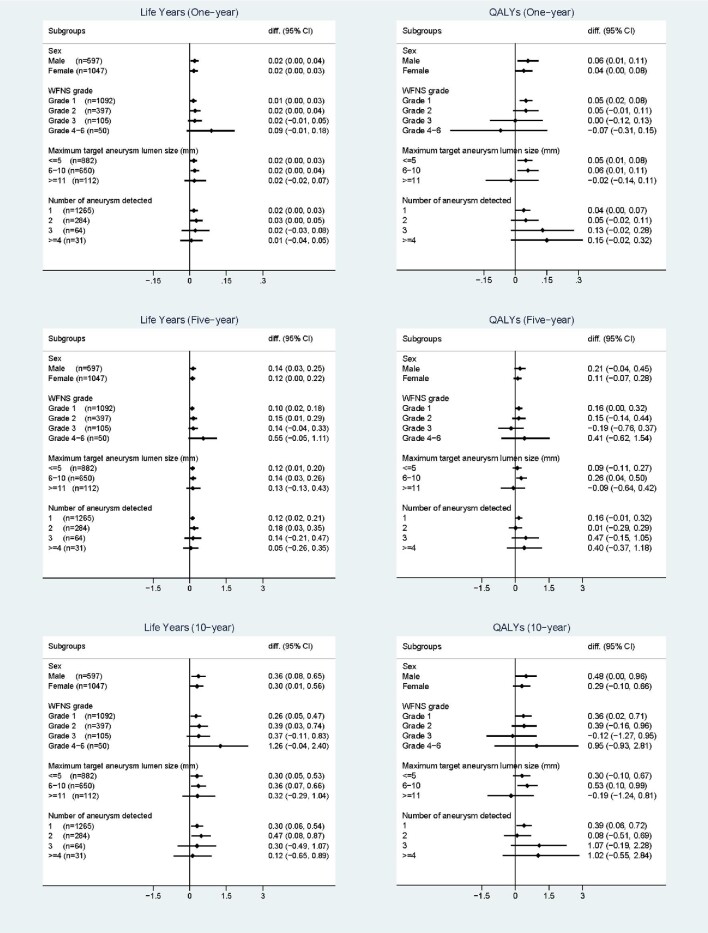
Subgroup analysis: differences in estimated life years and QALYs between treatment groups (endovascular coiling minus neurosurgery clipping) in different patient subgroups.

## DISCUSSION

In this study, based on a large, multicenter, randomized controlled trial, we looked at trends in survival, dependency, and HRQoL over 10 yr in patients with ruptured intracranial aneurysms who were treated with either endovascular coiling or neurosurgery clipping. The results show that the survival and independence status as well as overall HRQoL was better in the coiling group throughout the follow-up. The 10-yr mean difference in QALYs between the 2 treatment groups was 0.36 yr, with a third of this gained coming from differences in HRQoL alone.

This is the first study, to our knowledge, using a large randomized controlled trial to investigate whether there is a difference in HRQoL between SAH patients operated with endovascular coiling compared with neurosurgery clipping. Previous studies on this topic were mostly based on observational studies or in a nonrandomized fashion,^14,15^ which were limited by potential confounding factors.

The long and frequent follow-up in this study help to generate a profile of the dependency and HRQoL trajectory for SAH survivors. Both the dependency status and HRQoL were found to be considerable impaired at 2 mo after the intervention, then it gradually improved over time, until year 4 when recovery stabilizes. The pattern of changes in HRQoL for SAH survivors found in this study is similar to previous studies.^14,19^

Life years and QALYs are 2 frequently used long-term health outcomes in economics evaluations.^20^ Life years can capture the accumulated survival gain from the intervention and provide more information to compare with other long-term clinical endpoints such as all-cause mortality. The QALYs further incorporate the impact on both the quantity and HRQoL and provides a more comprehensive summary measure for a disease like stroke which has both a high mortality rate and long-term impact on HRQoL. Using QALYs as an outcome measure in this context, therefore, has the advantage of capturing the impact of specific SAH-related events after original intervention, such as rebleeds and retreatments over time.

The mRS for SAH survivors at year 10 was reported in the 2015 ISAT paper as a dichotomous variable (0-2 vs 3-5), with no statistically significant difference detected between the 2 groups.^11^ However, a recent study has suggested that retaining the full ordinal range of the mRS better relates to long-term outcomes of ischemic stroke than dichotomy. Such study recommended the use of full ordinal range of mRS because any movement in mRS can be meaningful and it would be inappropriate to assume that some states are equivalent concerning long-term outcomes.^21^ We found that a statistically significant difference in the mRS distribution existed in most of the follow-up years including year 10 using the full ordinal range, with a clear pattern of a higher proportion of patients reporting on more independent levels in the coiling group. This difference was not captured in previous study using the mRS as a dichotomous variable because the shifting from mRS level 2 to level 0 was not captured, with patients reporting mRS level 0 to 2 grouped as a single category.

The endorsement of applying coiling in clinical practice for patients judged to be technically amenable to both treatments was made based on its ability to reduce death and disability.^8^ ISAT was a selected population of SAH patients with mostly good grade patients with small anterior circulation aneurysms, which are the most common patients presenting for treatment. However, not all patients with ruptured aneurysms are suitable for both treatments and there may be anatomical and clinical considerations that may favor a surgical approach.

### Limitations

This study was subject to some limitations. We were unable to collect the EQ-5D-3L measurements immediately after the intervention. We used the 2-mo HRQoL for it, which may lead to a slight overestimate of the calculation of QALYs. Over time, significantly younger patients at trial entry in both groups had missing mRS and EQ-5D-3L responses with marginally more missing data in the neurosurgery compared with the endovascular group. If the additional missing data observed in the neurosurgery arm were due to younger patients not returning the questionnaires because of ill health, then our complete case analysis could have underestimated the 10-yr QALY gained. Our scenario analysis using last value carried forward, nevertheless, assumed on average lower EQ-5D-3L scores for the neurosurgery arm from year 5 onwards and showed a similar 10-yr QALY gained as the complete case analysis. This indicates that influence on missing data over time on our base case results were likely to be minor.

We were only able to follow the UK cohort for 10 yr because of funding and logistical constraints. The non-UK cohort had fewer pretreatment rebleeds than the UK cohort because of more rapid intervention in the non-UK cohort, particularly in the clipping patients.^12^ This may have resulted in a greater difference in the observed dichotomous outcomes in the UK than in the non-UK patients than would have been observed in the whole cohort. The odds ratio (OR) of a good outcome, excluding pretreatment rebleeds, was significant in the coiling group at 1-yr OR 0.77 (0.67-0.92) but did not reach significance at 5 yr OR 0.88 (0.77-1.02).^12^

## CONCLUSION

In conclusion, in this study based on a large, multicenter, randomized controlled trial, we looked at trends of survival, dependency, and HRQoL over 10 yr in patients with ruptured intracranial aneurysms who were treated with either endovascular coiling or neurosurgery clipping. The results show that the survival and independence status as well as overall HRQoL was better in the endovascular coiling group throughout the period of follow-up. Patients in the endovascular coiling group were estimated to have improvement in life years and QALYs in both short and long term.

### Funding

The ISAT was funded by a Medical Research Council grant (number G0700479, ended in June 2013; previous grant number G9401611, which ended in June 2007). The research was supported by the National Institute for Health Research Oxford Biomedical Research Centre.

### Disclosures

The views expressed are those of the author(s) and not necessarily those of the National Health Service, the National Institute for Health Research, or the Department of Health. The authors have no personal, financial, or institutional interest in any of the drugs, materials, or devices described in this article. Molyneux is a consultant for Sequent Medical (clinical case adjudication and clinical study advice) and provides expert witness evidence in cases of SAH. Kerr provides expert witness evidence in cases of SAH.

## Supplementary Material

nyaa454_Supplemental_Files

## Notes

**Supplemental Digital Content 1. Text.** Expanded methods: outcomes.

**Supplemental Digital Content 2. Text.** Expanded methods: handling missing values for mRS and EQ-5D-3L responses.

**Supplemental Digital Content 3. Text.** Expanded methods: all-cause mortality multivariate Cox model.

**Supplemental Digital Content 4. Text.** Expanded methods: methods to calculate life years and QALYs and to decompose QALYs gain.

**Supplemental Digital Content 5. Figure.** CONSORT PRO flow diagram.

**Supplemental Digital Content 6. Table.** Baseline characteristics at trial entry for patients with complete mRS and missing data at follow-up point in the endovascular group.

**Supplemental Digital Content 7. Table.** Baseline characteristics at trial entry for patients with complete EQ-5D-3L and missing data at follow-up point in the endovascular group.

**Supplemental Digital Content 8. Table.** Baseline characteristics at trial entry for patients with complete mRS and missing data at follow-up point in the neurosurgery group.

**Supplemental Digital Content 9. Table.** Baseline characteristics at trial entry for patients with complete EQ-5D-3L and missing data at follow-up point in the neurosurgery group.

**Supplemental Digital Content 10. Table.** Ten-year follow-up on survival and the mRS for SAH survivors.

**Supplemental Digital Content 11. Table.** Ten-year follow-up on EQ-5D-3L utilities for SAH survivors, by treatment groups.

**Supplemental Digital Content 12. Table.** Distribution (n, %) of EQ-5D-3L responses across the dimensions for available data up to 10 yr follow-up.

## COMMENT

The authors of this work present the long-term results evaluating survival, dependency, and health-related quality of life in a subset of patients from the International Subarachnoid Aneurysm Trial. From 1644 patients enrolled from the United Kingdom, a median follow-up of 12.8 years was secured. The authors retrieved quality of life details through a postal questionnaire at two months post-treatment and yearly. After an observed initial period of impairment in functional status and quality of life, there was a gradual improvement with subsequent stabilization of functionality at year four. There was a consistently higher proportion of asymptomatic patients and a lower death rate in the endovascular group throughout follow-up. The authors found a higher proportion of all-cause mortality in the surgical clipping group compared with patients treated with endovascular coiling (H.R. 1.35; 95% CI: 1.08–1.68). The absolute gain in QALY of 0.36 years favored those treated with endovascular coils. These data are no less than quite informative for physicians and serves as a high-quality source of evidence for patient counseling. The trends presented herein demonstrate that the immediate clinical benefits found in the endovascular group of the ISAT trial, translate into a significantly higher proportion of patients with functional independence and better health-related quality of life over an observation period of 10 years. It is still important to emphasize that these patients do not represent all of the ruptured aneurysm patients, and surgical clipping is yet a highly valuable treatment of certain type of ruptured aneurysms.

**Santiago Gomez-Paz**

**Ajith J. Thomas**

**Christopher S. Ogilvy**

*Boston, Massachusetts*
