# Supplementary material for: Survival, Dependency, and Health-Related Quality of Life in Patients With Ruptured Intracranial Aneurysm: 10-Year Follow-up of the United Kingdom Cohort of the International Subarachnoid Aneurysm Trial
Source: Neurosurgery. 2020 Oct 19;88(2):252–60. doi: 10.1093/neuros/nyaa454 (PMC7803435; doi:10.1093/neuros/nyaa454)
Supplement: nyaa454_Supplemental_Files [file nyaa454_supplemental_files.zip › SDC1.docx]

**Supplemental Digital Content 1. Text. Expanded methods: Outcomes**

We assessed rates of death, dependence, and HRQoL after neurosurgical clipping or endovascular coiling over a period of 10 years. Dependency status was measured by the patient-reported modified Rankin Scale (mRS) questionnaire. The scale includes 7 different levels measuring the degree of dependency running from no symptoms (level 0) to death (level 6). HRQoL was measured by the 3-level version of EQ-5D (EQ-5D-3L) questionnaire developed by the EuroQol Group, in which patients were required to report if there was any problems (none, some, or unable/extreme) in 5 domains (mobility, self-care, usual activities, pain/discomfort and anxiety/depression) respectively. The responses from the questionnaire were then converted into a utility value ranging from −0.59 (worse than death) to 1 (perfect health) using UK population value set to represent a patient’s overall quality of life.
