# Supplementary material for: Survival, Dependency, and Health-Related Quality of Life in Patients With Ruptured Intracranial Aneurysm: 10-Year Follow-up of the United Kingdom Cohort of the International Subarachnoid Aneurysm Trial
Source: Neurosurgery. 2020 Oct 19;88(2):252–60. doi: 10.1093/neuros/nyaa454 (PMC7803435; doi:10.1093/neuros/nyaa454)
Supplement: nyaa454_Supplemental_Files [file nyaa454_supplemental_files.zip › SDC2.docx]

**Supplemental Digital Content 2. Text. Expanded methods: Handling missing values for mRS and EQ-5D-3L responses**

There were missing values for some responses to the mRS and the EQ-5D-3L, while complete information was available about mortality during follow-up through linkage to the Office for National Statistics. Because of this and to minimise the impact of over representation of deaths, the following adjustments were made in the descriptive analysis:

- For the mRS, when reporting the distribution of mRS responses, the proportion of reporting mRS level 6 (dead) were calculated as number of deaths divided by the total number of patients in the cohort; the proportion of reporting mRS level 0 to 5 were calculated by multiplying the probability of being alive and probability of reporting on each individual level of the mRS among patients who reported a mRS at that time point;
- For the EQ-5D-3L, when calculating mean utility values at different time points, weights were generated to avoid bias caused by over sampling of death. For dead cases the weight equals 1. For alive patients with a non-missing EQ-5D-3L utility, the weight equals the total number of alive patients divided by the number of patients with a non-missing EQ-5D-3L utility at that time point.
