# Supplementary material for: Survival, Dependency, and Health-Related Quality of Life in Patients With Ruptured Intracranial Aneurysm: 10-Year Follow-up of the United Kingdom Cohort of the International Subarachnoid Aneurysm Trial
Source: Neurosurgery. 2020 Oct 19;88(2):252–60. doi: 10.1093/neuros/nyaa454 (PMC7803435; doi:10.1093/neuros/nyaa454)
Supplement: nyaa454_Supplemental_Files [file nyaa454_supplemental_files.zip › SDC3.docx]

**Supplemental Digital Content 3. Text. Expanded methods: All-cause mortality multivariate Cox model**

A multivariable Cox model was fitted for all-cause mortality with treatment group and patient baseline characteristics (age, sex, World Federation of Neurological Surgeons (WFNS) grade, maximum target aneurysm lumen size, number of aneurysm detected) as predictors. The proportional hazard assumption was examined using Schoenfeld residuals. Interactions between the treatment and other risk factors were checked by adding in interaction terms between them. Survival curves were generated based on the estimated Cox model and then used to calculate life years as area under the curve.
