# Supplementary material for: Survival, Dependency, and Health-Related Quality of Life in Patients With Ruptured Intracranial Aneurysm: 10-Year Follow-up of the United Kingdom Cohort of the International Subarachnoid Aneurysm Trial
Source: Neurosurgery. 2020 Oct 19;88(2):252–60. doi: 10.1093/neuros/nyaa454 (PMC7803435; doi:10.1093/neuros/nyaa454)
Supplement: nyaa454_Supplemental_Files [file nyaa454_supplemental_files.zip › SDC4.docx]

**Supplemental Digital Content 4. Text. Expanded methods: Methods to calculate life years and quality-adjusted life years (QALYs) and to decompose QALYs gain**

Step 1. Using the estimated Cox model, estimate survival probabilities at 2 month and annually up to 10 years based on individual risk factors on patient level

Step 2. Using the survival probabilities to estimate life years for each time period (0-2 month, 2 month- 1 year, 1-2 year, etc.) on patient level

Step 3. Estimate average life years for each time period (0-2 month, 2 month- 1 year, 1-2 year, etc.) for the whole sample and for the two treatment groups

Step 4. Estimate average EQ-5D-3L utility scores at each time point (2 month and annually to 10 years) for the whole sample and for the two treatment groups.

Step 5. Multiplying results from step 3 and 4 to calculate average QALYs for each time period (0-2 month, 2 month- 1 year, 1-2 year, etc.) for the whole sample and for the two treatment groups. A mean utility value of the starting and ending point is used for each time period. For example, the average 2nd year QALYs = average life years in 1-2 year * (average utility at year 1 + average utility at year 2)/2

Step 6. Add up average life years and QALYs in different time periods to get one-year, five-year, and ten-year life years and QALYs for the whole sample and for the two treatment groups, as well as differences between the two groups

Step 7. Bootstrap and repeat step 1-6 for 1000 times to get confidence intervals for results in step 6.

To decompose ten-year QALYs:

In step 5, when estimating average QALYs for each time period for the two treatment groups, use the group specific average life years estimated in step 3 and the whole sample average EQ-5D-3L utility scores (instead of the group specific ones) in step 4 to do the calculation. The remaining steps are the same.
