# Supplementary material for: Survival, Dependency, and Health-Related Quality of Life in Patients With Ruptured Intracranial Aneurysm: 10-Year Follow-up of the United Kingdom Cohort of the International Subarachnoid Aneurysm Trial
Source: Neurosurgery. 2020 Oct 19;88(2):252–60. doi: 10.1093/neuros/nyaa454 (PMC7803435; doi:10.1093/neuros/nyaa454)
Supplement: nyaa454_Supplemental_Files [file nyaa454_supplemental_files.zip › SDC5.docx]

**Supplemental Digital Content 5. Figure. CONSORT PRO flow diagram**

Endovascular treatment (n= 809)

Neurosurgery (n= 835)

Allocated to neurosurgery (n= 1070)

Allocated to endovascular treatment (n= 1073)

## Enrolment

Randomized (n= 2143)

Assessed for eligibility (n= 9559)

Excluded (n= 7416)

♦  Declined to participate (n= 671)

♦  Other reasons (n= 6745)

## Allocation

## Analysis

## (UK cohort)

## Follow-up on patient-reported outcomes

(Complete all-cause mortality data provided during follow-up through data linkage with the Office for National Statistics)

- Two months after intervention

2 missing modified Rankin Scale

91 missing health-related quality of life

- Five years after intervention

62 missing modified Rankin Scale

117 missing health-related quality of life

- Ten years after intervention

129 missing modified Rankin Scale

156 missing health-related quality of life

- Two months after intervention

4 missing modified Rankin Scale

119 missing health-related quality of life

- Five years after intervention

80 missing modified Rankin Scale

146 missing health-related quality of life

- Ten years after intervention

179 missing modified Rankin Scale

195 missing health-related quality of life
