# Supplementary material for: Survival, Dependency, and Health-Related Quality of Life in Patients With Ruptured Intracranial Aneurysm: 10-Year Follow-up of the United Kingdom Cohort of the International Subarachnoid Aneurysm Trial
Source: Neurosurgery. 2020 Oct 19;88(2):252–60. doi: 10.1093/neuros/nyaa454 (PMC7803435; doi:10.1093/neuros/nyaa454)
Supplement: nyaa454_Supplemental_Files [file nyaa454_supplemental_files.zip › SDC6.docx]

**Supplemental Digital Content 6. Table. Baseline characteristics at trial entry for patients with complete mRS and missing data at follow-up point in the endovascular group**

|  | Complete mRS at 2 months  (n = 807) | Missing mRS at 2 months  (n = 2) | p-value** | Complete mRS at 5 years  (n = 747) | Missing mRS at 5 years  (n = 62) | p-value | Complete mRS at 10 years  (n = 680) | Missing mRS at 10 years  (n = 129) | p-value |
| --- | --- | --- | --- | --- | --- | --- | --- | --- | --- |
| Age (years)* | 52 (43-60) | 62 (60-64) | 0.15 | 52 (44-60) | 48 (39-57) | 0.01 | 53 (45-60) | 46 (38-53) | <0.001 |
| Sex |  |  |  |  |  |  |  |  |  |
| Female | 516 (64%) | 1 (50%) | 0.68 | 483 (65%) | 34 (55%) | 0.12 | 438 (64%) | 79 (61%) | 0.49 |
| Male | 291 (36%) | 1 (50%) |  | 264 (35%) | 28 (45%) |  | 242 (36%) | 50 (39%) |  |
| WFNS grade |  |  |  |  |  |  |  |  |  |
| 1 | 546 (68%) | 0 (0%) | 0.14 | 505 (68%) | 41 (66%) | 0.42 | 467 (69%) | 79 (61%) | 0.07 |
| 2 | 184 (23%) | 1 (50%) |  | 167 (22%) | 18 (29%) |  | 143 (21%) | 42 (33%) |  |
| 3 | 49 (6%) | 1 (50%) |  | 49 (7%) | 1 (2%) |  | 44 (6%) | 6 (5%) |  |
| 4 | 18 (2%) | 0 (0%) |  | 17 (2%) | 1 (2%) |  | 17 (3%) | 1 (1%) |  |
| 5 | 5 (1%) | 0 (0%) |  | 4 (1%) | 1 (2%) |  | 5 (1%) | 0 (0%) |  |
| 6 | 5 (1%) | 0 (0%) |  | 5 (1%) | 0 (0%) |  | 4 (1%) | 1 (1%) |  |
| Maximum target aneurysm lumen size (mm) |  |  |  |  |  |  |  |  |  |
| ≤5 | 430 (53%) | 1 (50%) | 0.91 | 391 (52%) | 40 (65%) | 0.17 | 357 (53%) | 74 (57%) | 0.42 |
| 6-10 | 321 (40%) | 1 (50%) |  | 304 (41%) | 18 (29%) |  | 273 (40%) | 49 (38%) |  |
| ≥11 | 56 (7%) | 0 (0%) |  | 52 (7%) | 4 (6%) |  | 50 (7%) | 6 (5%) |  |
| Number of aneurysms detected |  |  |  |  |  |  |  |  |  |
| 1 | 614 (76%) | 1 (50%) | <0.001 | 566 (76%) | 49 (79%) | 0.95 | 515 (76%) | 100 (78%) | 0.65 |
| 2 | 145 (18%) | 0 (0%) |  | 135 (18%) | 10 (16%) |  | 121 (18%) | 24 (19%) |  |
| 3 | 32 (4%) | 0 (0%) |  | 30 (4%) | 2 (3%) |  | 28 (4%) | 4 (3%) |  |
| ≥4 | 16 (2%) | 1 (50%) |  | 16 (2%) | 1 (2%) |  | 16 (2%) | 1 (1%) |  |
| Time between subarachnoid haemorrhage and randomisation (days)* | 2 (1-5) | 14 (7-21) | 0.04 | 2 (1-5) | 3 (2-6) | 0.15 | 2 (1-5) | 3 (2-5) | 0.13 |
| WFNS = World Federation of Neurological Surgeons; * Median (IQR); **Wilcoxon rank test for continuous measures, and Pearson’s chi-squared for categorical measures | | | | | | | | | |
