# Supplementary material for: Survival, Dependency, and Health-Related Quality of Life in Patients With Ruptured Intracranial Aneurysm: 10-Year Follow-up of the United Kingdom Cohort of the International Subarachnoid Aneurysm Trial
Source: Neurosurgery. 2020 Oct 19;88(2):252–60. doi: 10.1093/neuros/nyaa454 (PMC7803435; doi:10.1093/neuros/nyaa454)
Supplement: nyaa454_Supplemental_Files [file nyaa454_supplemental_files.zip › SDC7.docx]

**Supplemental Digital Content 7. Table. Baseline characteristics at trial entry for patients with complete EQ-5D-3L and missing data at follow-up point in the endovascular group**

|  | Complete EQ-5D-3L at 2 months  (n = 718) | Missing EQ-5D-3L at 2 months  (n = 91) | p-value** | Complete EQ-5D-3L at 5 years  (n = 692) | Missing EQ-5D-3L at 5 years  (n = 117) | p-value | Complete EQ-5D-3L at 10 years  (n = 653) | Missing EQ-5D-3L at 10 years  (n = 156) | p-value |
| --- | --- | --- | --- | --- | --- | --- | --- | --- | --- |
| Age (years)* | 52 (43-59) | 53 (44-62) | 0.55 | 53 (44-61) | 48 (39-54) | <0.001 | 53 (45-61) | 46 (38-53) | <0.001 |
| Sex |  |  |  |  |  |  |  |  |  |
| Female | 458 (64%) | 59 (65%) | 0.84 | 456 (66%) | 61 (52%) | 0.004 | 420 (64%) | 97 (62%) | 0.62 |
| Male | 260 (36%) | 32 (35%) |  | 236 (34%) | 56 (48%) |  | 233 (36%) | 59 (38%) |  |
| WFNS grade |  |  |  |  |  |  |  |  |  |
| 1 | 494 (69%) | 52 (57%) | 0.10 | 467 (67%) | 79 (68%) | 0.84 | 450 (69%) | 96 (62%) | 0.14 |
| 2 | 155 (22%) | 30 (33%) |  | 156 (23%) | 29 (25%) |  | 137 (21%) | 48 (31%) |  |
| 3 | 42 (6%) | 8 (9%) |  | 45 (7%) | 5 (4%) |  | 41 (6%) | 9 (6%) |  |
| 4 | 17 (2%) | 1 (1%) |  | 15 (2%) | 3 (3%) |  | 16 (2%) | 2 (1%) |  |
| 5 | 5 (1%) | 0 (0%) |  | 4 (1%) | 1 (1%) |  | 5 (1%) | 0 (0%) |  |
| 6 | 5 (1%) | 0 (0%) |  | 5 (1%) | 0 (0%) |  | 4 (1%) | 1 (1%) |  |
| Maximum target aneurysm lumen size (mm) |  |  |  |  |  |  |  |  |  |
| ≤5 | 386 (54%) | 45 (49%) | 0.45 | 359 (52%) | 72 (62%) | 0.08 | 341 (52%) | 90 (58%) | 0.28 |
| 6-10 | 285 (40%) | 37 (41%) |  | 281 (41%) | 41 (35%) |  | 263 (40%) | 59 (38%) |  |
| ≥11 | 47 (7%) | 9 (10%) |  | 52 (8%) | 4 (3%) |  | 49 (8%) | 7 (4%) |  |
| Number of aneurysms detected |  |  |  |  |  |  |  |  |  |
| 1 | 554 (77%) | 61 (67%) | 0.08 | 525 (76%) | 90 (77%) | 0.55 | 496 (76%) | 119 (76%) | 0.30 |
| 2 | 122 (17%) | 23 (25%) |  | 122 (18%) | 23 (20%) |  | 113 (17%) | 32 (21%) |  |
| 3 | 29 (4%) | 3 (3%) |  | 30 (4%) | 2 (2%) |  | 28 (4%) | 4 (3%) |  |
| ≥4 | 13 (2%) | 4 (4%) |  | 15 (2%) | 2 (2%) |  | 16 (2%) | 1 (1%) |  |
| Time between subarachnoid haemorrhage and randomisation (days)* | 2 (1-5) | 2 (1-6) | 0.98 | 2 (1-5) | 3 (2-5) | 0.12 | 2 (1-5) | 3 (2-5) | 0.22 |
| WFNS = World Federation of Neurological Surgeons; * Median (IQR); **Wilcoxon rank test for continuous measures, and Pearson’s chi-squared for categorical measures | | | | | | | | | |
