# Supplementary material for: Survival, Dependency, and Health-Related Quality of Life in Patients With Ruptured Intracranial Aneurysm: 10-Year Follow-up of the United Kingdom Cohort of the International Subarachnoid Aneurysm Trial
Source: Neurosurgery. 2020 Oct 19;88(2):252–60. doi: 10.1093/neuros/nyaa454 (PMC7803435; doi:10.1093/neuros/nyaa454)
Supplement: nyaa454_Supplemental_Files [file nyaa454_supplemental_files.zip › SDC8.docx]

**Supplemental Digital Content 8. Table. Baseline characteristics at trial entry for patients with complete mRS and missing data at follow-up point in the neurosurgery group**

|  | Complete mRS at 2 months  (n = 831) | Missing mRS at 2 months  (n = 4) | p-value** | Complete mRS at 5 years  (n = 755) | Missing mRS at 5 years  (n = 80) | p-value | Complete mRS at 10 years  (n = 656) | Missing mRS at 10 years  (n = 179) | p-value |
| --- | --- | --- | --- | --- | --- | --- | --- | --- | --- |
| Age (years)* | 52 (44-60) | 43 (39-52) | 0.22 | 53 (44-60) | 48 (41-58) | 0.02 | 53 (45-61) | 48 (40-55) | <0.001 |
| Sex |  |  |  |  |  |  |  |  |  |
| Female | 528 (64%) | 2 (50%) | 0.57 | 483 (64%) | 47 (59%) | 0.36 | 428 (65%) | 102 (57%) | 0.04 |
| Male | 303 (36%) | 2 (50%) |  | 272 (36%) | 33 (41%) |  | 228 (35%) | 77 (43%) |  |
| WFNS grade |  |  |  |  |  |  |  |  |  |
| 1 | 542 (65%) | 4 (100%) | 0.71 | 491 (65%) | 55 (69%) | 0.64 | 426 (65%) | 120 (67%) | 0.15 |
| 2 | 212 (26%) | 0 (0%) |  | 192 (25%) | 20 (25%) |  | 163 (25%) | 49 (27%) |  |
| 3 | 55 (7%) | 0 (0%) |  | 50 (7%) | 5 (6%) |  | 45 (7%) | 10 (6%) |  |
| 4 | 16 (2%) | 0 (0%) |  | 16 (2%) | 0 (0%) |  | 16 (2%) | 0 (0%) |  |
| 5 | 0 (0%) | 0 (0%) |  | 0 (0%) | 0 (0%) |  | 0 (0%) | 0 (0%) |  |
| 6 | 6 (1%) | 0 (0%) |  | 6 (1%) | 0 (0%) |  | 6 (1%) | 0 (0%) |  |
| Maximum target aneurysm lumen size (mm) |  |  |  |  |  |  |  |  |  |
| ≤5 | 448 (54%) | 3 (75%) | 0.67 | 401 (53%) | 50 (63%) | 0.27 | 353 (54%) | 98 (55%) | 0.60 |
| 6-10 | 327 (39%) | 1 (25%) |  | 302 (40%) | 26 (33%) |  | 256 (39%) | 72 (40%) |  |
| ≥11 | 56 (7%) | 0 (0%) |  | 52 (7%) | 4 (5%) |  | 47 (7%) | 9 (5%) |  |
| Number of aneurysms detected |  |  |  |  |  |  |  |  |  |
| 1 | 647 (78%) | 3 (75%) | 0.14 | 587 (78%) | 63 (79%) | 0.58 | 519 (79%) | 131 (73%) | 0.27 |
| 2 | 139 (17%) | 0 (0%) |  | 125 (17%) | 14 (18%) |  | 105 (16%) | 34 (19%) |  |
| 3 | 31 (4%) | 1 (25%) |  | 31 (4%) | 1 (1%) |  | 23 (4%) | 9 (5%) |  |
| ≥4 | 14 (2%) | 0 (0%) |  | 12 (2%) | 2 (3%) |  | 9 (1%) | 5 (3%) |  |
| Time between subarachnoid haemorrhage and randomisation (days)* | 3 (1-6) | 3 (3-4) | 0.74 | 3 (1-5) | 3 (1-6) | 0.83 | 3 (1-6) | 2 (1-5) | 0.12 |
| WFNS = World Federation of Neurological Surgeons; * Median (IQR); **Wilcoxon rank test for continuous measures, and Pearson’s chi-squared for categorical measures | | | | | | | | | |
