# Supplementary material for: Survival, Dependency, and Health-Related Quality of Life in Patients With Ruptured Intracranial Aneurysm: 10-Year Follow-up of the United Kingdom Cohort of the International Subarachnoid Aneurysm Trial
Source: Neurosurgery. 2020 Oct 19;88(2):252–60. doi: 10.1093/neuros/nyaa454 (PMC7803435; doi:10.1093/neuros/nyaa454)
Supplement: nyaa454_Supplemental_Files [file nyaa454_supplemental_files.zip › SDC9.docx]

**Supplemental Digital Content 9. Table. Baseline characteristics at trial entry for patients with complete EQ-5D-3L and missing data at follow-up point in the neurosurgery group**

|  | Complete EQ-5D-3L at 2 months  (n = 716) | Missing EQ-5D-3L at 2 months  (n = 119) | p-value** | Complete EQ-5D-3L at 5 years  (n = 689) | Missing EQ-5D-3L at 5 years  (n = 146) | p-value | Complete EQ-5D-3L at 10 years  (n = 640) | Missing EQ-5D-3L at 10 years  (n = 195) | p-value |
| --- | --- | --- | --- | --- | --- | --- | --- | --- | --- |
| Age (years)* | 52 (44-59) | 53 (45-61) | 0.60 | 53 (45-60) | 48 (41-55) | <0.001 | 53 (45-61) | 48 (40-56) | <0.001 |
| Sex |  |  |  |  |  |  |  |  |  |
| Female | 450 (63%) | 80 (67%) | 0.36 | 455 (66%) | 75 (51%) | <0.001 | 415 (65%) | 115 (59%) | 0.14 |
| Male | 266 (37%) | 39 (33%) |  | 234 (34%) | 71 (49%) |  | 225 (35%) | 80 (41%) |  |
| WFNS grade |  |  |  |  |  |  |  |  |  |
| 1 | 475 (66%) | 71 (60%) | 0.29 | 446 (65%) | 100 (68%) | 0.38 | 415 (65%) | 131 (67%) | 0.26 |
| 2 | 179 (25%) | 33 (28%) |  | 179 (26%) | 33 (23%) |  | 161 (25%) | 51 (26%) |  |
| 3 | 44 (6%) | 11 (9%) |  | 43 (6%) | 12 (8%) |  | 43 (7%) | 12 (6%) |  |
| 4 | 12 (2%) | 4 (3%) |  | 15 (2%) | 1 (1%) |  | 16 (3%) | 0 (0%) |  |
| 5 | 0 (0%) | 0 (0%) |  | 0 (0%) | 0 (0%) |  | 0 (0%) | 0 (0%) |  |
| 6 | 6 (1%) | 0 (0%) |  | 6 (1%) | 0 (0%) |  | 5 (1%) | 1 (1%) |  |
| Maximum target aneurysm lumen size (mm) |  |  |  |  |  |  |  |  |  |
| ≤5 | 392 (55%) | 59 (50%) | 0.51 | 368 (53%) | 83 (57%) | 0.68 | 346 (54%) | 105 (54%) | 0.93 |
| 6-10 | 278 (39%) | 50 (42%) |  | 273 (40%) | 55 (38%) |  | 250 (39%) | 78 (40%) |  |
| ≥11 | 46 (6%) | 10 (8%) |  | 48 (7%) | 8 (5%) |  | 44 (7%) | 12 (6%) |  |
| Number of aneurysms detected |  |  |  |  |  |  |  |  |  |
| 1 | 551 (77%) | 99 (83%) | 0.27 | 542 (79%) | 108 (74%) | 0.16 | 508 (79%) | 142 (73%) | 0.22 |
| 2 | 122 (17%) | 17 (14%) |  | 108 (16%) | 31 (21%) |  | 101 (16%) | 38 (19%) |  |
| 3 | 29 (4%) | 3 (3%) |  | 29 (4%) | 3 (2%) |  | 22 (3%) | 10 (5%) |  |
| ≥4 | 14 (2%) | 0 (0%) |  | 10 (1%) | 4 (3%) |  | 9 (1%) | 5 (3%) |  |
| Time between subarachnoid haemorrhage and randomisation (days)* | 3 (1-6) | 3 (1-5) | 0.68 | 3 (1-5) | 3 (2-6) | 0.28 | 3 (1-6) | 2 (1-5) | 0.27 |
| WFNS = World Federation of Neurological Surgeons; * Median (IQR); **Wilcoxon rank test for continuous measures, and Pearson’s chi-squared for categorical measures | | | | | | | | | |
