# Supplementary material for: Survival, Dependency, and Health-Related Quality of Life in Patients With Ruptured Intracranial Aneurysm: 10-Year Follow-up of the United Kingdom Cohort of the International Subarachnoid Aneurysm Trial
Source: Neurosurgery. 2020 Oct 19;88(2):252–60. doi: 10.1093/neuros/nyaa454 (PMC7803435; doi:10.1093/neuros/nyaa454)
Supplement: nyaa454_Supplemental_Files [file nyaa454_supplemental_files.zip › SDC10.docx]

**Supplemental Digital Content 10. Table. Ten-year follow-up on survival and the modified Rankin Scale (mRS) for subarachnoid hemorrhage survivors**

|  | **Endovascular (n=809)** | | | | | **Neurosurgery (n=835)** | | | | | **Chi-square for mRS (P value)** |
| --- | --- | --- | --- | --- | --- | --- | --- | --- | --- | --- | --- |
|  | **No. Dead** | **No. Alive** | **Independence by mRS** | | | **No. Dead** | **No. Alive** | **Independence by mRS** | | |  |
| **2 month** | 47 | 762 | 0 | 141/760 | (18·6%) | 67 | 768 | 0 | 103/764 | (13·5%) | **31·26 (<0·001)** |
|  |  |  | 1 | 243/760 | (32·0%) |  |  | 1 | 213/764 | (27·9%) |  |
|  |  |  | 2 | 218/760 | (28·7%) |  |  | 2 | 199/764 | (26·0%) |  |
|  |  |  | 3 | 94/760 | (12·4%) |  |  | 3 | 163/764 | (21·3%) |  |
|  |  |  | 4 | 22/760 | (2·9%) |  |  | 4 | 36/764 | (4·7%) |  |
|  |  |  | 5 | 42/760 | (5·5%) |  |  | 5 | 50/764 | (6·5%) |  |
|  |  |  |  |  |  |  |  |  |  |  |  |
| **Year 1** | 56 | 753 | 0 | 188/751 | (25·0%) | 83 | 752 | 0 | 144/747 | (19·3%) | **14·18 (0·015)** |
|  |  |  | 1 | 231/751 | (30·8%) |  |  | 1 | 228/747 | (30·5%) |  |
|  |  |  | 2 | 193/751 | (25·7%) |  |  | 2 | 189/747 | (25·3%) |  |
|  |  |  | 3 | 98/751 | (13·0%) |  |  | 3 | 120/747 | (16·1%) |  |
|  |  |  | 4 | 25/751 | (3·3%) |  |  | 4 | 37/747 | (5·0%) |  |
|  |  |  | 5 | 16/751 | (2·1%) |  |  | 5 | 29/747 | (3·9%) |  |
|  |  |  |  |  |  |  |  |  |  |  |  |
| **Year 2** | 58 | 751 | 0 | 228/734 | (31·1%) | 91 | 744 | 0 | 193/705 | (27·4%) | 10·14 (0·071) |
|  |  |  | 1 | 214/734 | (29·2%) |  |  | 1 | 191/705 | (27·1%) |  |
|  |  |  | 2 | 168/734 | (22·9%) |  |  | 2 | 165/705 | (23·4%) |  |
|  |  |  | 3 | 76/734 | (10·4%) |  |  | 3 | 110/705 | (15·6%) |  |
|  |  |  | 4 | 28/734 | (3·8%) |  |  | 4 | 29/705 | (4·1%) |  |
|  |  |  | 5 | 20/734 | (2·7%) |  |  | 5 | 17/705 | (2·4%) |  |
|  |  |  |  |  |  |  |  |  |  |  |  |
| **Year 3** | 64 | 745 | 0 | 231/709 | (32·6%) | 106 | 729 | 0 | 183/673 | (27·2%) | **11·39 (0·044)** |
|  |  |  | 1 | 208/709 | (29·3%) |  |  | 1 | 191/673 | (28·4%) |  |
|  |  |  | 2 | 149/709 | (21·0%) |  |  | 2 | 168/673 | (25·0%) |  |
|  |  |  | 3 | 78/709 | (11·0%) |  |  | 3 | 100/673 | (14·9%) |  |
|  |  |  | 4 | 26/709 | (3·7%) |  |  | 4 | 17/673 | (2·5%) |  |
|  |  |  | 5 | 17/709 | (2·4%) |  |  | 5 | 14/673 | (2·1%) |  |
|  |  |  |  |  |  |  |  |  |  |  |  |
| **Year 4** | 71 | 738 | 0 | 228/690 | (33·0%) | 111 | 724 | 0 | 191/652 | (29·3%) | 5·11 (0·403) |
|  |  |  | 1 | 196/690 | (28·4%) |  |  | 1 | 175/652 | (26·8%) |  |
|  |  |  | 2 | 141/690 | (20·4%) |  |  | 2 | 156/652 | (23·9%) |  |
|  |  |  | 3 | 85/690 | (12·3%) |  |  | 3 | 95/652 | (14·6%) |  |
|  |  |  | 4 | 23/690 | (3·3%) |  |  | 4 | 19/652 | (2·9%) |  |
|  |  |  | 5 | 17/690 | (2·5%) |  |  | 5 | 16/652 | (2·5%) |  |
|  |  |  |  |  |  |  |  |  |  |  |  |
| **Year 5** | 76 | 733 | 0 | 245/671 | (36·5%) | 116 | 719 | 0 | 181/639 | (28·3%) | **13·88 (0·016)** |
|  |  |  | 1 | 182/671 | (27·1%) |  |  | 1 | 188/639 | (29·4%) |  |
|  |  |  | 2 | 122/671 | (18·2%) |  |  | 2 | 151/639 | (23·6%) |  |
|  |  |  | 3 | 80/671 | (11·9%) |  |  | 3 | 87/639 | (13·6%) |  |
|  |  |  | 4 | 24/671 | (3·6%) |  |  | 4 | 20/639 | (3·1%) |  |
|  |  |  | 5 | 18/671 | (2·7%) |  |  | 5 | 12/639 | (1·9%) |  |
|  |  |  |  |  |  |  |  |  |  |  |  |
| **Year 6** | 88 | 721 | 1 | 232/630 | (36·8%) | 125 | 710 | 0 | 175/604 | (29·0%) | **17·95 (0·003)** |
|  |  |  | 1 | 180/630 | (28·6%) |  |  | 1 | 155/604 | (25·7%) |  |
|  |  |  | 2 | 118/630 | (18·7%) |  |  | 2 | 160/604 | (26·5%) |  |
|  |  |  | 3 | 64/630 | (10·2%) |  |  | 3 | 81/604 | (13·4%) |  |
|  |  |  | 4 | 19/630 | (3·0%) |  |  | 4 | 20/604 | (3·3%) |  |
|  |  |  | 5 | 17/630 | (2·7%) |  |  | 5 | 13/604 | (2·2%) |  |
|  |  |  |  |  |  |  |  |  |  |  |  |
| **Year 7** | 103 | 706 | 0 | 240/611 | (39·3%) | 145 | 690 | 0 | 184/578 | (31·8%) | **16·22 (0·006)** |
|  |  |  | 1 | 168/611 | (27·5%) |  |  | 1 | 149/578 | (25·8%) |  |
|  |  |  | 2 | 106/611 | (17·3%) |  |  | 2 | 132/578 | (22·8%) |  |
|  |  |  | 3 | 62/611 | (10·1%) |  |  | 3 | 79/578 | (13·7%) |  |
|  |  |  | 4 | 17/611 | (2·8%) |  |  | 4 | 24/578 | (4·2%) |  |
|  |  |  | 5 | 18/611 | (2·9%) |  |  | 5 | 10/578 | (1·7%) |  |
|  |  |  |  |  |  |  |  |  |  |  |  |
| **Year 8** | 113 | 696 | 0 | 230/583 | (39·5%) | 159 | 676 | 0 | 185/557 | (33·2%) | 10·03 (0·074) |
|  |  |  | 1 | 150/583 | (25·7%) |  |  | 1 | 134/557 | (24·1%) |  |
|  |  |  | 2 | 101/583 | (17·3%) |  |  | 2 | 126/557 | (22·6%) |  |
|  |  |  | 3 | 73/583 | (12·5%) |  |  | 3 | 74/557 | (13·3%) |  |
|  |  |  | 4 | 15/583 | (2·6%) |  |  | 4 | 24/557 | (4·3%) |  |
|  |  |  | 5 | 14/583 | (2·4%) |  |  | 5 | 14/557 | (2·5%) |  |
|  |  |  |  |  |  |  |  |  |  |  |  |
| **Year 9** | 125 | 684 | 0 | 216/557 | (38·8%) | 166 | 669 | 0 | 171/506 | (33·8%) | **11·73 (0·039)** |
|  |  |  | 1 | 153/557 | (27·5%) |  |  | 1 | 121/506 | (23·9%) |  |
|  |  |  | 2 | 96/557 | (17·2%) |  |  | 2 | 116/506 | (22·9%) |  |
|  |  |  | 3 | 60/557 | (10·8%) |  |  | 3 | 67/506 | (13·2%) |  |
|  |  |  | 4 | 16/557 | (2·9%) |  |  | 4 | 22/506 | (4·3%) |  |
|  |  |  | 5 | 16/557 | (2·9%) |  |  | 5 | 9/506 | (1·8%) |  |
|  |  |  |  |  |  |  |  |  |  |  |  |
| **Year 10** | 135 | 674 | 0 | 227/545 | (41·7%) | 178 | 657 | 0 | 162/478 | (33·9%) | **13·31 (0·021)** |
|  |  |  | 1 | 137/545 | (25·1%) |  |  | 1 | 107/478 | (22·4%) |  |
|  |  |  | 2 | 85/545 | (15·6%) |  |  | 2 | 107/478 | (22·4%) |  |
|  |  |  | 3 | 68/545 | (12·5%) |  |  | 3 | 75/478 | (15·7%) |  |
|  |  |  | 4 | 17/545 | (3·1%) |  |  | 4 | 18/478 | (3·8%) |  |
|  |  |  | 5 | 11/545 | (2·0%) |  |  | 5 | 9/478 | (1·9%) |  |

Bold figures indicate significant differences at 5% level
