# Supplementary material for: Survival, Dependency, and Health-Related Quality of Life in Patients With Ruptured Intracranial Aneurysm: 10-Year Follow-up of the United Kingdom Cohort of the International Subarachnoid Aneurysm Trial
Source: Neurosurgery. 2020 Oct 19;88(2):252–60. doi: 10.1093/neuros/nyaa454 (PMC7803435; doi:10.1093/neuros/nyaa454)
Supplement: nyaa454_Supplemental_Files [file nyaa454_supplemental_files.zip › SDC11.docx]

**Supplemental Digital Content 11. Table. Ten-year follow-up on EQ-5D-3L utilities for subarachnoid hemorrhage survivors, by treatment groups**

|  | **Endovascular** | | **Neurosurgery** | | **t-test for mean utility difference (P value)** |
| --- | --- | --- | --- | --- | --- |
|  | **n** | **EQ-5D-3L utility***  **Mean (SDs)** | **n** | **EQ-5D-3L utility***  **Mean (SDs)** |  |
| **2 months** | 671 | 0·681 (0·32) | 649 | 0·640 (0·32) | **2·302 (0·022**) |
| **Year 1** | 716 | 0·728 (0·30) | 689 | 0·699 (0·31) | 1·733 (0·083) |
| **Year 2** | 670 | 0·739 (0·30) | 631 | 0·720 (0·30) | 1·112 (0·266) |
| **Year 3** | 644 | 0·743 (0·31) | 595 | 0·746 (0·28) | -0·140 (0·888) |
| **Year 4** | 633 | 0·748 (0·31) | 586 | 0·757 (0·28) | -0·519 (0·604) |
| **Year 5** | 616 | 0·751 (0·31) | 573 | 0·752 (0·30) | -0·030 (0·976) |
| **Year 6** | 572 | 0·767 (0·30) | 542 | 0·743 (0·29) | 1·388 (0·165) |
| **Year 7** | 576 | 0·765 (0·31) | 510 | 0·740 (0·30) | 1·333 (0·183) |
| **Year 8** | 554 | 0·771 (0·29) | 518 | 0·748 (0·30) | 1·266 (0·206) |
| **Year 9** | 527 | 0·763 (0·31) | 480 | 0·759 (0·28) | 0·220 (0·826) |
| **Year 10** | 518 | 0·760 (0·30) | 462 | 0·758 (0·29) | 0·124 (0·901) |

Bold figures indicate significant differences at 5% level
