# Supplementary material for: Survival, Dependency, and Health-Related Quality of Life in Patients With Ruptured Intracranial Aneurysm: 10-Year Follow-up of the United Kingdom Cohort of the International Subarachnoid Aneurysm Trial
Source: Neurosurgery. 2020 Oct 19;88(2):252–60. doi: 10.1093/neuros/nyaa454 (PMC7803435; doi:10.1093/neuros/nyaa454)
Supplement: nyaa454_Supplemental_Files [file nyaa454_supplemental_files.zip › SDC12.docx]

**Supplemental Digital Content 12. Table. Distribution (n, %) of EQ-5D-3L responses across the dimensions for available data up to 10 years follow-up**

|  | **Mobility** | | **Self-care** | | **Usual Activities** | | **Pain** | | **Anxiety** | | |
| --- | --- | --- | --- | --- | --- | --- | --- | --- | --- | --- | --- |
| **Follow-up point** | **Endovascular** | **Neurosurgery** | **Endovascular** | **Neurosurgery** | **Endovascular** | **Neurosurgery** | **Endovascular** | **Neurosurgery** | **Endovascular** | **Neurosurgery** | |
| ***2 months*** |  |  |  |  |  |  |  |  |  |  | |
| 1 | 432 (64%) | 374 (60%) | 537 (79%) | 476 (73%) | 273 (40%) | 253 (39%) | 291 (43%) | 242 (37%) | 336 (50%) | 285 (44%) | |
| 2 | 215 (32%) | 249 (35%) | 105 (16%) | 147 (22%) | 322 (48%) | 315 (48%) | 364 (54%) | 382 (59%) | 290 (43%) | 313 (48%) | |
| 3 | 30 (5%) | 35 (5%) | 35 (5%) | 34 (5%) | 82 (12%) | 90 (14%) | 20 (3%) | 29 (4%) | 47 (7%) | 52 (8%) | |
| χ^2^ (p-value) | **6·78** | **0·034** | **10·39** | **0·006** | 0·94 | 0·625 | **6·23** | **0·044** | 4·92 | 0·085 | |
| ***1 year*** |  |  |  |  |  |  |  |  |  |  | |
| 1 | 487 (68%) | 454 (65%) | 594 (83%) | 550 (79%) | 410 (57%) | 764 (54%) | 384 (53%) | 347 (50%) | 360 (50%) | 319 (46%) | |
| 2 | 218 (30%) | 232 (33%) | 107 (15%) | 129 (18%) | 258 (36%) | 543 (38%) | 302 (42%) | 313 (45%) | 313 (44%) | 316 (46%) | |
| 3 | 14 (2%) | 13 (2%) | 19 (3%) | 20 (3%) | 52 (7%) | 60 (8%) | 32 (4%) | 31 (4%) | 44 (6%) | 56 (8%) | |
| χ^2^ (p-value) | 1·348 | 0·510 | 3·458 | 0·177 | 5·709 | 0·058 | 1·568 | 0·456 | 3·451 | 0·178 | |
| ***2 year*** |  |  |  |  |  |  |  |  |  |  | |
| 1 | 456 (68%) | 416 (65%) | 560 (83%) | 511 (80%) | 415 (61%) | 354 (56%) | 385 (57%) | 332 (52%) | 369 (55%) | 695 (53%) | |
| 2 | 207 (31%) | 209 (33%) | 93 (14%) | 108 (17%) | 222 (33%) | 232 (36%) | 254 (38%) | 280 (44%) | 257 (38%) | 519 (40%) | |
| 3 | 12 (2%) | 11 (2%) | 20 (3%) | 17 (3%) | 38 (6%) | 51 (8%) | 34 (5%) | 22 (3%) | 46 (7%) | 91 (7%) | |
| χ^2^ (p-value) | 0·728 | 0·695 | 2·560 | 0·278 | 5·862 | 0·053 | **6·597** | **0·037** | 1·556 | 0·459 | |
| ***3 year*** |  |  |  |  |  |  |  |  |  |  | |
| 1 | 433 (67%) | 397 (66%) | 535 (83%) | 491 (82%) | 389 (60%) | 341 (57%) | 383 (59%) | 339 (57%) | 352 (55%) | 318 (53%) | |
| 2 | 204 (32%) | 195 (33%) | 93 (14%) | 97 (16%) | 219 (34%) | 233 (39%) | 233 (36%) | 231 (39%) | 251 (39%) | 247 (41%) | |
| 3 | 9 (1%) | 7 (1%) | 18 (3%) | 11 (2%) | 37 (6%) | 25 (4%) | 28 (4%) | 25 (4%) | 42 (7%) | 32 (5%) | |
| χ^2^ (p-value) | 0·241 | 0·887 | 1·889 | 0·389 | 4·217 | 0·121 | 0·924 | 0·630 | 1·256 | 0·534 | |
| ***4 year*** |  |  |  |  |  |  |  |  |  |  | |
| 1 | 425 (67%) | 397 (67%) | 521 (82%) | 480 (81%) | 400 (63%) | 347 (59%) | 384 (61%) | 344 (59%) | 363 (57%) | 329 (56%) | |
| 2 | 200 (31%) | 188 (32%) | 98 (15%) | 96 (16%) | 202 (32%) | 213 (36·16%) | 216 (34%) | 226 (38%) | 232 (37%) | 227 (39%) | |
| 3 | 11 (2%) | 6 (1%) | 17 (3%) | 13 (2%) | 33 (5%) | 29 (5%) | 34 (5%) | 18 (3%) | 38 (6%) | 33 (6%) | |
| χ^2^ (p-value) | 1·147 | 0·564 | 0·431 | 0·806 | 2·585 | 0·275 | 5·624 | 0·060 | 0·494 | 0·781 | |
| ***5 year*** |  |  |  |  |  |  |  |  |  |  | |
| 1 | 421 (68%) | 383 (66%) | 508 (82%) | 478 (83%) | 387 (62%) | 336 (58%) | 391 (63%) | 343 (60%) | 359 (58%) | 334 (58%) | |
| 2 | 189 (30%) | 187 (32%) | 97 (16%) | 86 (15%) | 203 (33%) | 208 (36%) | 197 (32%) | 207 (36%) | 217 (35%) | 212 (37%) | |
| 3 | 10 (2%) | 10 (2%) | 16 (3%) | 14 (2%) | 30 (5%) | 34 (6%) | 31 (5%) | 26 (5%) | 43 (7%) | 27 (5%) | |
| χ^2^ (p-value) | 0·474 | 0·789 | 0·165 | 0·921 | 2·439 | 0·295 | 2·281 | 0·320 | 2·846 | 0·241 | |
| ***6 year*** |  |  |  |  |  |  |  |  |  |  | |
| 1 | 398 (69%) | 340 (63%) | 488 (85%) | 449 (82%) | 379 (66%) | 308 (57%) | 351 (61%) | 319 (59%) | 360 (63%) | 307 (56%) | |
| 2 | 171 (30%) | 197 (36%) | 76 (13%) | 82 (15%) | 171 (30%) | 212 (39%) | 187 (33%) | 202 (37%) | 190 (33%) | 207 (38%) | |
| 3 | 8 (1%) | 7 (1%) | 13 (2%) | 14 (3%) | 28 (5%) | 25 (5%) | 36 (6%) | 24 (4%) | 25 (4%) | 30 (6%) | |
| χ^2^ (p-value) | 5·495 | 0·064 | 0·976 | 0·614 | **10·936** | **0·004** | 3·758 | 0·153 | 4·539 | 0·103 | |
| ***7 year*** |  |  |  |  |  |  |  |  |  |  | |
| 1 | 400 (69%) | 326 (63%) | 491 (85%) | 424 (82%) | 380 (66%) | 311 (60%) | 371 (64%) | 299 (58%) | 364 (63) | 304 (59%) | |
| 2 | 168 (29%) | 185 (36%) | 70 (12%) | 81 (16%) | 166 (29%) | 174 (34%) | 169 (29%) | 185 (36%) | 189 (33%) | 190 (37%) | |
| 3 | 11 (2%) | 8 (2%) | 17 (3%) | 14 (3%) | 33 (6%) | 34 (7%) | 38 (7%) | 30 (6%) | 25 (4%) | 21 (4%) | |
| χ^2^ (p-value) | 5·573 | 0·062 | 2·833 | 0·243 | 3·826 | 0·148 | 5·670 | 0·059 | 2·115 | 0·347 | |
| ***8 year*** |  |  |  |  |  |  |  |  |  |  |  |
| 1 | 376 (67%) | 336 (64%) | 478 (86%) | 428 (82%) | 364 (65%) | 316 (60%) | 343 (62%) | 310 (60%) | 342 (61%) | 312 (60%) |  |
| 2 | 175 (31%) | 181 (34%) | 66 (12%) | 82 (16%) | 167 (30%) | 178 (34%) | 187 (34%) | 184 (35%) | 188 (34%) | 179 (34%) |  |
| 3 | 7 (1%) | 8 (2%) | 15 (3%) | 14 (3%) | 28 (5%) | 31 (6%) | 26 (5%) | 25 (5%) | 27 (5%) | 28 (5%) |  |
| χ^2^ (p-value) | 1·411 | 0·494 | 3·396 | 0·183 | 2·828 | 0·243 | 0·439 | 0·803 | 0·273 | 0·872 |  |
| ***9 year*** |  |  |  |  |  |  |  |  |  |  |  |
| 1 | 348 (65%) | 292 (60%) | 440 (83%) | 396 (83%) | 332 (62%) | 288 (60%) | 346 (65%) | 286 (59%) | 334 (63%) | 287 (60%) |  |
| 2 | 173 (33%) | 185 (38%) | 79 (15%) | 78 (16%) | 168 (32%) | 171 (35%) | 162 (30%) | 174 (36%) | 170 (32%) | 182 (38%) |  |
| 3 | 11 (2%) | 7 (1%) | 14 (3%) | 9 (2%) | 32 (6%) | 24 (5%) | 24 (5%) | 21 (4%) | 27 (5%) | 12 (2%) |  |
| χ^2^ (p-value) | 3·932 | 0·140 | 0·951 | 0·622 | 1·931 | 0·381 | 3·767 | 0·152 | **7·283** | **0·026** |  |
| ***10 year*** |  |  |  |  |  |  |  |  |  |  |  |
| 1 | 339 (64%) | 281 (60%) | 436 (83%) | 379 (81%) | 329 (62%) | 271 (58%) | 323 (61%) | 277 (59%) | 333 (63%) | 290 (63%) |  |
| 2 | 180 (34%) | 180 (38%) | 79 (15%) | 77 (16%) | 167 (32%) | 170 (36%) | 176 (33%) | 171 (37%) | 168 (32%) | 158 (34%) |  |
| 3 | 7 (1%) | 7 (2%) | 12 (2%) | 12 (3%) | 32 (6%) | 26 (6%) | 27 (5%) | 19 (4%) | 27 (5%) | 15 (3%) |  |
| χ^2^ (p-value) | 2·049 | 0·359 | 0·516 | 0·773 | 2·524 | 0·283 | 1·490 | 0·475 | 2·450 | 0·294 |  |

Bold figures indicate significant differences at 5% level
